# Parental Monitoring, Parent-Adolescent Communication, and Adolescents’ Trust in Their Parents in China

**DOI:** 10.1371/journal.pone.0134730

**Published:** 2015-08-13

**Authors:** Liuhua Ying, Fengling Ma, Huahua Huang, Xiaolin Guo, Chuansheng Chen, Fen Xu

**Affiliations:** 1 Department of Psychology, Zhejiang Sci-Tech University, Hangzhou, Zhejiang, PRC; 2 National Innovation Center for Assessment of Basic Education Quality, Beijing Normal University, Beijing, PRC; 3 Department of Psychology and Social Behavior, University of California Irvine, Irvine, California, United States of America; University of New South Wales, AUSTRALIA

## Abstract

**Purpose:**

Trust is an important aspect of interpersonal relationships, but little is known about adolescents’ interpersonal trust. The aim of the present study was to examine the associations among parental monitoring, parent-adolescent communication, and adolescents’ trust in their parents in China.

**Methods:**

Data in this study were collected as part of the cross-sectional study of children in China. 3349 adolescents (female 48.6%, age range of 12–15 years) were randomly selected from 35 secondary schools in April, 2009 and administered to the Adolescent Interpersonal Trust Scale, the Parental Monitoring Scale, and the Parent-Adolescent Communication Scale.

**Results:**

Adolescents’ trust in their parents was positively related to parental monitoring and parent-adolescent communication. Furthermore, parent-adolescent communication mediated the association between parental monitoring and adolescents’ trust in their parents. The mediation model fit data of both genders and three age groups equally well.

**Conclusions:**

Parental monitoring and parent-adolescent communication play an importance role in fostering adolescents’ trust in their parents.

## Introduction

Trust within parent-child relationship refers to a confident belief in each other’s reliability, emotional trustworthiness, and honesty [1].Children’s trust in their parents plays an important role in normal socio-emotional development. For example, attachment theory proposes that trust in caregivers’ availability, dependability, and responsiveness affects the children’s current and future social function [2, 3]. In Erikson’s (1950) developmental theory, establishing basic trust with the caregiver is a key development task in early childhood and is central to how adolescent identity crisis is resolved [4].Empirical studies have also shown that adolescents who perceive a strong mutual trust with their parents tend to engage in fewer high-risk behaviors [5, 6, 7], to feel less loneliness [8], to report a higher level of parent-adolescent relational qualities [9], and to show better psychosocial adjustment [10]. Less is known, however, about what parenting factors may lead to adolescents’ trust in their parents[6, 9]. In the current study, we examined the role of parental monitoring in adolescents’ trust in their parents and how it may be mediated by parent-adolescent communication.

Parental monitoring is a set of parenting behaviors that parents are engaged in to gain information about their children’s activities, whereabouts, and friendships[11, 12].Effective parental monitoring should structure the child’s environment and actively track the child’s activities. Furthermore, previous studies showed that compared with their counterparts, Chinese parents would exert greater supervision and control in child-rearing in addition to provide care to the child [13]. As children enter adolescence, they spend an increasing amount of time outside the family and in company of peers. Parental monitoring becomes less reliant on supervision and control and includes more autonomy-granting. Researchers have found that parental autonomy granting, rather than parental control, would promote adolescents’ honesty and facilitates mutual trust[14]. Indeed, by striking the right balance between autonomy/individuation and relatedness/connectedness, parents and adolescents can maintain trust in and warmth with each other. When the balance is upset, less-than-ideal outcomes appear. For example, Kerr and Stattin (2000) showed that when children view parental monitoring as invasive and overly controlling, they may react to this intrusion by engaging in even more delinquent behaviors that their parents could not penetrate or control [15].

In addition to parents’ active tracking and surveillance, which may be interpreted as intrusions, another form of parental monitoring—parental knowledge—has also been studied[15, 16, 17]. Moreover, parental knowledge has been viewed as a determinant of parent-adolescent trust. For instance, a previous study showed that parental knowledge of the child’s prior delinquent behavior as well as the child’s activities in school and during free time is closely linked to trust[6]. In addition, knowledge of daily activities that the child freely supplied was strongly linked to trust. Finkenauer and other colleagues (2002)[18]found that adolescents’ willingness to tell parents about their activities is associated with adolescents’ perceptions of better relationships with parents and parents’ greater responsiveness to their teens.

Although both parental monitoring in general and parental knowledge in particular have been linked toa higher level of parent-adolescent trust, little is known about what intermediate processes may explain the relations. We propose that one such mediating process is parent-adolescent communication because both parental monitoring and parental knowledge have been linked to parent-adolescent communications, which in turn has been associated with trust. Communication is key to build and strengthen connectedness and intimacy between parents and their children [6, 18]. Open communication between parents and adolescents may contribute to affective qualities of parent-child relationships[9, 19, 20]. A previous study showed that the frequency and quality of communication between parents and their children were related to adolescents’ perceptions of parental trustworthiness [19]. In addition, parental behaviors also influence the pattern of communication between parents and adolescents. For instance, Metzger and other colleagues (2012) [21] found that parents who had positive attitudes toward the efficacy of monitoring reported higher levels of open communication, whereas parents who did not put a premium on respecting their child’s privacy showed increased parent-adolescent conflict and reduced adolescent disclosure.

Developmental changes of adolescents’ interpersonal or social trust have been found in the previous studies [22, 23]. For example, Hestenes (1996) [23] found that early and middle adolescents were similar in their willingness to depend on mothers and fathers. With increasing age, adolescents were less likely to share private thoughts, feelings, and secrets with their parents. Previous studies have also shown some gender differences in adolescents’ trust in their parents. During middle childhood, daughters are more willing than sons to place trust in their mothers [23, 24].

Based on the above literature review, we proposed that parental monitoring (including parental knowledge) would be positively associated with adolescents’ trust in their parents and that parental-adolescent communication would mediate this association. We also investigated whether these associations varied by age and gender. These hypotheses were tested with a national sample of adolescents in China.

## Method

### Participants and procedure

Data in this study were collected as part of the cross-sectional study of children in China (for full detail; http:www.cddata-china.org.html.). The study was approved by ethics committees of Beijing Normal University, China and informed written consent was obtained from participants and their guardians. In the current study, 3394 adolescents were randomly selected from 35 secondary schools in April, 2009. 45 of 3394 participants declined from the current study, yielding a response rate of 98.7%. To investigate the potential impact of attrition, we tested the differences in demographic variables (i.e., age, grade, and gender) and main study variables (i.e., parent monitoring, parent-adolescent communication, and adolescents’ trust in their parents) between the 3349 participants and the 45 participants who had incomplete data. Results showed no significant differences in these variables between the two groups. The 3349 participants (female 48.6%, age range of 12–15 years) were about evenly distributed across three grade levels: 1092 7th graders (*M* age = 13.51 years, *SD* = 0.83), 1136 8th graders (*M* age = 14.48 years, *SD* = 0.78), and 1121 9th graders (*M* age = 15.38 years, *SD* = 0.70).

### Measures

#### Parental monitoring

Parental monitoring was assessed by the Parental Monitoring Scale (PMS), which was modified from the Regulation Scale for Chinese Adolescents (RSCA) [25] and the Parental Autonomy Granting Scale (PAGS) [26]. It consisted of 17 items and three subscales: parental knowledge, parental negative control, and autonomy granting. The parental knowledge subscale measures the extent to which parents were knowledgeable about adolescents’ whereabouts and activities (e.g., do your parents always start a conversation with you about your free times?). The parental control subscale measures the extent to which parental imposed restrictions on adolescents’ activities (e.g., do your parents always require that you tell them where you are at night?). The autonomy granting subscale measures the degree to which parents grant adolescents’ autonomy (e.g., do you have to get parent permission to stay out late on a weekday evening?). Participants responded to the items on a 5-scale ranging from 0 (not true at all) to 4 (true nearly all the time) with higher scores reflecting high levels of parent monitoring. In the current study, the scores of the three subscales were computed and used in the analysis as continuous variables. Previous studies of Chinese adolescents showed that the original PMS had good psychometric properties [27]. In the current study, results of confirmatory factor analysis found an acceptable fit of a model with the three subscales (χ^2^ = 2282.76, *df* = 116, χ^2^/df = 19.68, RMSEA = 0.05, CFI = 0.90, TLI = 0.89). Cronbach alphas of the three subscales ranged from .67 to .76.

#### Parent-adolescent communication

Parent-adolescent communication was assessed by using the Adolescents’ Communication with Parents Scale [28]. The 19-item self-report instrument was designed to assess the quality of parent-adolescent communication. Participants were asked how well the descriptions (e.g., “I often share my views of something with parents”) fit their interactions with their parents using a 5-point scale (0 = “not at all”, 1 = “somewhat”, 2 = “rather well”, 3 = “well”, and 4 = “very well”). Higher ACPS scores indicated a good and open parent-child communication. In the current study, the total score of the scale was computed and used in the analysis as continuous variables. Previous study showed that the original PCS has demonstrated good psychometric properties in samples of Chinese adolescents [28]. In the current study, results of confirmatory factor analysis found an acceptable fit of a model with the three subscales (χ^2^ = 2597.81, *df* = 146, χ^2^/df = 17.79, RMSEA = 0.07, CFI = 0.88, TLI = 0.86).Cronbach alpha of the scale was .88.

#### Children’s trust in parents scale

Adolescents’ trust in their parents was measured by the Children’s Trust in Parents Scale (CTPS) developed by Li, Li, and Zou (2008) [29]. The 14-item instrument contains three domains: parent dependability, honesty, and sharing secrets. Each item was scored on a 5-point scale ranging from 0 (not true at all) to 4 (true nearly all the time). Higher scores indicated a greater degree of adolescents’ trust in their parents. In the current study, the total score of the scale was computed and used in the analysis as continuous variables. Previous study showed that the original PCS has good psychometric properties [29]. In the current study, confirmatory factor analysis of the current sample found an acceptable fit of a model with the three subscales (χ^2^ = 1436.23, *df* = 74, χ^2^/*df* = 19.41, RMSEA = 0.071, CFI = 0.91, TLI = 0.89). Cronbach alphas of the three subscales ranged from .72 to .78.

### Data analysis

First, Multivariate liner analyses were conducted to evaluate gender and grade-level differences in adolescents’ trust in their parents. Then Pearson correlations were calculated to examine the associations between parental monitoring, parent-adolescent communication, and adolescents’ trust in their parents. Third, the mediated model was tested with the AMOS7.0 software using the Maximum Likelihood (ML) iteration procedure. The degree of model fit was assessed using the following fit indices: the Chi-square, the normed-chi-square tests (Normedχ2) [30], the Tucker–Lewis Index (TLI) [31], the Comparative Fit Index (CFI) [32], and the Root Mean Square Error of Approximation (RMSEA) [33]. Models were considered as fitting the data if TLI and CFI were > .95, and RMSEA was < .06 [34]. Finally, we conducted multi-group comparisons of the mediated model to investigate whether the model fit both genders and three grade levels equally well. Specifically, the unconstrained model where the coefficient paths were allowed to vary across gender and levels of grade was compared to a model where the coefficient paths were constrained to be equal. The test of the difference of Chi-square was used to evaluate the difference in modal fit.

## Results

### Gender and grade-level differences in adolescents’ trust in parents

Analyses of variance were conducted for adolescents’ trust in their parents (i.e., the total score and three subscales of adolescents’ trust in parents) with gender and grade as the between-subject factors. For *the total score of the CTPS* there were significant effects of gender (*F*_*(1*, *3343)*_ = 13.49, *p* < .01) and grade (*F*_*(2*, *3343)*_ = 7.49, *p* < .01).Female adolescents had a higher level of trust in their parents than male adolescents. Adolescents at grades 7 (*M* = 3.53, *SD* = .76) and 9 (*M* = 3.52, *SD* = .74) had a higher level of trust in their parents than adolescents at grade 8 (*M* = 3.42, *SD* = .72), but adolescents at grades 7 and 9 did not differ from each other.

For *parent dependability*, there was a significant effect of gender (*F*_*(1*, *3343)*_ = 10.11, *p* < .01). Female adolescents (*M* = 3.95, *SD* = .81) reported a higher level of parent dependability than male adolescents (*M* = 3.86, *SD* = .80). There were no significant grade and grade-by-gender interaction effects. For *adolescent honesty*, there were significant effects of gender (*F*_*(1*, *3343)*_ = 36.11, *p* < .01) and grade (*F*_*(2*, *3343)*_ = 12.51, *p*< 0.01). Female adolescents (*M* = 3.17, *SD* = 1.00) reported a higher level of honesty than male adolescents (*M* = 2.94, *SD* = .93).Adolescents at grade 7 (*M* = 3.19, *SD* = .98) reported a higher level of honesty than adolescents at grades 8(*M* = 3.00, *SD* = .95) and 9 (*M* = 3.02, *SD* = .97), but adolescents at grades8 and 9 did not differ from each other. There was no significant interaction between gender and grade. For *sharing secrets*, there was a significant effect of grade (*F*_*(2*, *3343)*_ = 8.33, *p* < .01). Adolescents at grades 7 (*M* = 3.57, *SD* = .95) and 9 (*M* = 3.59, *SD* = .91) reported a higher level of sharing secrets than adolescents at grade 8 (*M* = 3.44, *SD* = .96), but adolescents at grades 7 and 9 did not differ from each other. There were no significant gender and gender-by-grade effects.

### Correlations among parental monitoring, parent-adolescent communication, and adolescents’ trust in their parents

Table 1 shows the correlations among parental monitoring, parent-adolescent communication, and adolescents’ trust in their parents. Results showed that adolescents’ communication with parents had significant correlations with the scores of the total trust scale as well as its three subscales (i.e., parent dependability, honesty, and sharing secrets).The correlation coefficients ranged from .56 to .78. Two aspects of parental monitoring (i.e., parental knowledge and autonomy permission) were positively and significantly correlated with adolescents’ trust in their parents, *r*’s ranging from .33to .54, whereas negative parental control was negatively and significantly correlated with adolescents’ trust in parents, *r*’s ranging from-.35to-.52. Finally, parent-adolescent communication was positively and significantly correlated with parental knowledge (*r* = .52) and autonomy permission (*r* = .50), but negatively with negative control (*r* = -.47).

**Table 1:** Correlations among main study variables.

Variables	1	2	3	4	5	6	7	8
1. CTPS total score	-	.83**	.86**	.78**	.78**	.52**	-.51**	.54**
2. CTPS-dependability		-	.61**	.50**	.62**	.47**	-.35**	.50**
3. CTPS-sharing secrets			-	.51**	.75**	.50**	-.44**	.45**
4. CTPS-honesty				-	.56**	.33**	-.52**	.41**
5. Parent-adolescent communication					-	.52**	-.47**	.50**
6. PMS-parental knowledge						-	-.17**	.50**
7. PMS-negative control							-	-.41**
8. PMS-autonomy granting								-

*Note*. PAGS: the Parental Autonomy Granting Scale; PMS: the Parental Monitoring Scale.

**< .01.

### Parent-adolescent communication mediated the association between parental monitoring and adolescents’ trust in their parents

**Fig 1:**
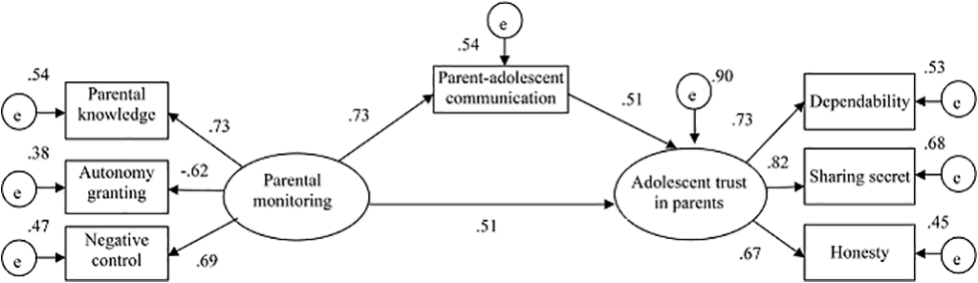
Parent-adolescent communication mediated the association between parental monitoring and adolescents’ trust in their parents. The figure revealed the positive association between parental monitoring and adolescents’ trust in their parents as well as the mediating role of parent-adolescent communication in this relationship.

To evaluate the mediation hypothesis, we tested a structural equation model that parental monitoring affected adolescents’ trust of their parents through parent-adolescent communication. Because gender and grade were significantly related to the main variables (i.e., parental monitoring, parent-adolescent communication and adolescents’ trust in parents), we also added them to the mediation model as two exogenous variables. Results showed that the model fit was acceptable (χ^2^ = 538.33, *df* = 24, *p*< .001; χ^2^/*df* = 22.43;RMSEA = .078; TLI = .942; CFI = .945), except that RMSEA was slightly higher than the threshold. As shown in Fig 1, parental monitoring was positively and significantly associated with adolescents’ trust in their parents. Furthermore, the indirect effect of parental monitoring on adolescents’ trust in their parents via parent-adolescent communication was significant.

Finally, we conducted multi-group comparisons of the mediated model to investigate whether the model fit both genders and three grade levels equally well. As shown in Table 2, results showed that the constrained model (which assumes no moderating influence of gender and grade) had no significantly poorer model fit relative to the unconstrained model (△χ^2^ = 7.99, △*df* = 7, *p*>.05). Similarly, constraining the path coefficient to be equal across three grade levels did not worsen model fit (△χ^2^ = 11.99, △*df* = 14, *p*>.05).

**Table 2:** The fit indices of the multigroup models.

Models	χ^2^	*df*	χ^2^/*df*	CFI	TLI	RMSEA
Grade 7	115.39***	11	10.49	.970	.942	.093
Grade 8	144.23***	11	13.11	.964	.930	.103
Grade 9	166.16***	11	15.11	.960	.921	.111
Male	167.44***	11	15.22	.972	.947	.093
Female	150.83***	11	13.71	.961	.939	.101

***p< .001.

## Discussion

In the current study, we examined the associations among parent monitoring, parent-adolescent communication, and Chinese adolescents’ trust in parents. Results showed that female adolescents reported a higher level of honesty and parent dependability. One possible explanation is that compared to male adolescents, female adolescents have a more frequent and intimate pattern of communication with their parents [35, 36], which may promote female adolescents’ trust in parents. Additionally, consistent with Henstenes (1996) study [23], the results showed that there was no significant grade difference in parent dependability. In terms of age differences, adolescents’ honesty and sharing secrets declined from grade 7 to grade 8, but sharing secrets restored to the initial level at grade 9. As suggested by the separation-individuation theory [37], early adolescents’ social and cognitive growth requires a sense of separation or individuation, which would lead to less honesty and less willingness to share secrets with their parents. During middle adolescence, however, disclosure would once again increase in order to attain a better relationship with their parents.

Results showed that parent-adolescent communication was positively related to Chinese adolescents’ trust in their parents. This finding was consistent with the previous results that open communication between parents and adolescents fostered mutual trust between them [9, 19, 20]. Communication between parents and children is usually considered as an important means of keeping their connectedness and intimacy [6, 18]. However, with increasing individuation, adolescents do not voluntarily share all information with their parents [12]. In this case, both parents and adolescents should adjust their communication behavior (e.g., decreasing secrecy by adolescents or increasing solicitation by parents) to attain a satisfying and mature relationship[36].

Similar to the previous studies [13, 14, 15], results of the current study showed that parental monitoring was positively related to adolescents’ trust in their parents. Although adolescence is a period marked by a greater desire for autonomy and independence, these results suggested that effective parental monitoring is an important factor of adolescent perception’s trust in parents. In addition to the direct effect, results of the present study suggested that parental monitoring also indirectly improved adolescents’ trust in their parents by affecting the quality of parent-adolescent communication. In other words, adolescents who reported higher levels of parental monitoring also reported a higher level of parent-adolescent communication, which then contributed to increased adolescents’ trust in their parents. Indeed, adolescents’ open communication with their parents is a key way of building an intimate parent-adolescent relationship [6, 18, 21]. Parenting behaviors that compromise adolescents’ feelings of connectedness and autonomy may have the effect of shutting down communication processes [38]. Taken together, parent-adolescent communication may play an important mediating role in the relationship between parental monitoring and adolescents’ trust in their parents.

Several limitations of the present study need to be mentioned. First, despite the large size of the sample, its cross-sectional design limited its ability to draw causal inference regarding the observed relationships. Thus, longitudinal studies are needed to help address the causality question among these variables. Second, the assessments of all variables relied on self-report and thus subject to self-reporting bias. Moreover, it is also possible that the associations between parental monitoring, parent-adolescent communication, and adolescents’ trust in parents might have been inflated due to the shared-method variance [39]. Thus, in future studies, it will be helpful to use multiple informants. Finally, because the data were collected in only a single country, the results may not generalize to other cultures.

Despite these shortcomings, the present findings offered important information on the role of parental monitoring in fostering adolescents’ trust in their parents. Moreover, this research provided new evidence regarding the mediating role of parent-adolescent communication in the association between parental monitoring and adolescents’ trust in their parents. Regarding clinical implications, the results suggested that the therapies for effective parental monitoring may have additional benefits of improving parent-adolescent communication and promoting adolescents’ trust in their parents. Intervention can also focus on parents’ communication with their adolescents.
